# Biomarkers in Community-Acquired Pneumonia (Cardiac and Non-Cardiac)

**DOI:** 10.3390/jcm9020549

**Published:** 2020-02-18

**Authors:** Raúl Méndez, Irene Aldás, Rosario Menéndez

**Affiliations:** 1 Pneumology Department, Hospital Universitario y Politécnico La Fe/Instituto de Investigación Sanitaria (IIS) La Fe, 46026 Valencia, Spain; Pneumology Department, Hospital Universitario y Politécnico La Fe, Avda, Fernando Abril Martorell 106, 46026 Valencia, Spain; rmendezalcoy@gmail.com; 2 University of Valencia, Medicine Faculty, 46026 Valencia, Spain; irene.aldas.criado@gmail.com; 3 Centro de Investigación Biomédica en Red Enfermedades Respiratorias (CIBERES), 28029 Madrid, Spain; 4 Pneumology Department, Hospital Germans Trias i Pujol, 08916 Badalona, Spain

**Keywords:** pneumonia, biomarkers, cardiac, cardiovascular

## Abstract

Community-acquired pneumonia (CAP) remains the first cause of morbidity and mortality worldwide due to infection. Several aspects such as severity and host response are related to its clinical course and outcome. Beyond the acute implications that the infection provokes in the host, pneumonia also has long-term negative consequences. Among them, cardiovascular complications and mortality are the most outstanding. Therefore, an adequate recognition and stratification of the risk of complications and mortality is crucial. Many biomarkers have been studied for these reasons, considering that each biomarker mirrors a different aspect. Moreover, the clinical application of many of them is still being deliberated because of their limitations and the heterogeneity of the disease. In this review, we examine some of the most relevant biomarkers that we have classified as cardiac and non-cardiac. We discuss some classic biomarkers and others that are considered novel biomarkers, which are mainly involved in cardiovascular risk.

## 1. Introduction

Community-acquired pneumonia (CAP), with an annual incidence of 3–11 cases per 1000 inhabitants in adult populations, is the first cause of sepsis [1,2]. Despite the progress made in recent decades, pneumonia continues to cause significant mortality worldwide, especially during childhood and in the elderly—mainly in countries with low average incomes. However, mortality in Europe and USA also remains unacceptably high, especially for severe pneumonia.

The impact of CAP does not only occur in the short-term during the acute phase of the infection. For some years now it has been shown that the morbidity and mortality mainly appeared during the first days, and it has been recently recognized that the increased risk for complications remains even years after the acute episode [3,4]. This occurs mostly as a consequence of the cardiovascular damage caused by the infection and the inflammatory host response, capable of leaving chronic sequelae that is predisposed to the occurrence of cardiovascular events and mortality [5]. The underlying pathophysiological mechanisms increase the cardiovascular risk, including inflammation, platelet activation, endothelial dysfunction, hypoxaemia, myocardial invasion, and specific pathogen-associated damage among others [5,6,7].

Stratification of the risk of mortality due to pneumonia, as well as identifying those patients most susceptible to complications in the short and long-term, are key in establishing preventive strategies and optimizing treatment [8]. Biomarkers in CAP could be useful for the diagnosis of infection [9,10], severity assessment [11], to monitor clinical response and antibiotic duration, and recently, to predict cardiovascular complications [12]. Many biomarkers have been studied for these purposes. The large number of biomarkers analysed reflects the complex pathophysiology of this disease and the heterogeneity of the host response. In this review, we briefly discuss some of the available biomarkers in CAP, including those reflecting cardiovascular damage. To this end, and for better understanding, we have classified the biomarkers as cardiac and non-cardiac. In Table 1, there is an overview of the biomarkers discussed in this issue, and Table 2 has a description of the studies.

## 2. Cardiac Biomarkers

### 2.1. Troponins

Cardiac troponins (cTns), cTnI and cTnT, are well known cardiac enzymes because they are sensitive and specific biomarkers of myocardial injury [105]. Their elevation is a consequence of myocardial damage. In CAP, myocardial damage may be produced directly by myocardium bacterial invasion, provoking cardiomyocyte apoptosis and necroptosis with a rise in cardiac enzymes [5]. Animal models show us that the cardiac tropism that some pathogens have, such as *Streptococcus pneumoniae*, explaining the appearance of CVE during the acute phase of CAP. These pneumococcal inclusions in the myocardium cause tissue damage that eventually develops into fibrous tissue. Other pathogenic mechanisms for myocardial damage in CAP are cardiac overload, reduced contractility, inflammation, and an oxygen supply-demand mismatch resulting in hypoxia over the myocardium [106,107,108]. In fact, AMI appears in around 8% (up to 15% in severe CAP) and it is associated with clinical failure and worse prognoses in CAP patients [3,109].

Troponin determination has been shown to be useful for short and long-term mortality prediction in septic and CAP patients [13,14,15,16]. Moreover, troponins could be used to predict cardiac events during and after CAP. Thus, patients with initial levels of troponin above 21.9 ng/L had a twofold-increased risk of CVE within the first 30 days [12]. However, at day 30 (when myocardial damaged is established and the fibrous tissue has already replaced dead cardiomyocytes) troponin T levels failed to predict CVE beyond that time. Raised levels of troponin in CAP have potential clinical implications because they may guide the prescribing of a cardiovascular rehabilitation program and treatment optimization. Moreover, it could be useful to select CAP patients at a high-risk of cardiovascular events in future trials.

### 2.2. Natriuretic Peptides

Natriuretic peptides are prohormones produced in numerous tissues. These peptides have multiple biological properties on diverse organs such as the regulation of homeostasis of body fluid volume and water/salt balance, and function as anti-remodelling factors in the heart [17]. The increase in its concentration is related to overload and cardiac stress. Among these biomarkers, midregional pro-atrial (A-type) natriuretic peptide (MR-proANP), N-terminal pro-brain (B-type) natriuretic peptide (NT-proBNP), and B-type natriuretic peptide (BNP) are the most studied.

The different peptides have shown good accuracy in guiding the site of care [18] and/or prognostic prediction in CAP [19,20,21,22,23]. The three biomarkers that have been studied demonstrate a similar predictive capacity for short and long-term mortality between them and with respect to the Pneumonia Severity Index (PSI) or CURB65 score, although not better results [24,25,26]. The combination of MR-proANP with these scores improves their long-term prognoses [27]. In comparison to inflammatory biomarkers, MR-proANP showed superiority in predicting short and long-term survival in CAP [28]. The usefulness of natriuretic peptides to predict CVE in CAP, specifically MR-proADM, was recently evaluated to identify patients at risk of early and long-term CVE. In that study, those patients with NT-proBNP levels above 1619 pg/mL at day 1 had more than a twofold-increased risk of early CVE, and levels above 315 pg/mL at day 30 day for CV events during one year [12].

### 2.3. Midregional-Proadrenomedullin

Adrenomedullin (ADM) is a strong vasodilatory prohormone with reliable measurement results being difficult to obtain. Instead, midregional-proadrenomedullin (MR-proADM) is a surrogate marker of ADM that is easier to measure [29]. ADM levels reveal excessive blood volume, inflammation, and cardiac dysfunction, so it could be considered both a cardiac and inflammatory biomarker. Specifically, the increased plasma concentration of ADM is present in patients with heart failure, acute myocardial infarction (AMI), or peripheral arterial occlusive disease [30,31,32]. In the case of MR-proADM, its biological activity is under study, but it seems to have pro and anti-inflammatory activity, inducing local inflammation in the injured heart tissue, but attenuating, exaggerated inflammation [33]. Now, it appears that besides a marker of cardiac damage and inflammation, it may have its own activity.

In CAP, MR-proADM has been found to be useful in improving the capacity of prognostic scales for decision-making in hospital and ICU admission [34,35]. This biomarker alone allows clinicians to estimate short and long-term survival rates in CAP, with a precision comparable to clinical scores, and its combination with the scores improves its accuracy [28,36,37,38,39]. In addition, the survival prediction power of pro-ADM is independent of the microbiological aetiology [40].

The prognosis in pneumonia is often conditioned by cardiovascular complications. To date, the utility of MR-proADM in CAP has only been evaluated for prognostic purposes. However, recently, this biomarker has been evaluated to predict cardiovascular events (CVE) in hospitalized CAP patients [12]. Menendez et al., in a multicentre study, showed that those patients with initial levels of MR-proADM above 1.2 nmol/L had a twofold-increased the risk of short-term CVE, adjusted for age, prior cardiovascular disease, respiratory failure and sepsis. Furthermore, levels above 0.83 nmol/L at day 30 also increased the risk of long-term CVE events (within one year of follow-up).

### 2.4. Endothelin-1

Endothelin-1 (ET-1), a peptide that mainly proceeds from endothelial cells, is a stronger vasoconstrictor compared to angiotensin II. ProET-1 is a more stable precursor of ET-1 and is easier to measure [41]. ET-1 causes decreased cardiac output and vasoconstriction in the coronary, pulmonary, renal, and splanchnic circulation [42]. Septic patients show elevated ET-1 plasma levels and its concentration is associated with morbidity and mortality [43]. In CAP, proET-1 levels at admission and their evolution have also demonstrated a good correlation with disease severity (assessed by prognostic scores) and are independently related to short and long-term mortality [28,44,45,46].

In hospitalized CAP, initial high levels (above 104 pmol/L) of proET-1 is a marker that expresses shear stress and exhibited good accuracy in predicting early CVE and levels above 70.7 pmol/L at day 30 for predicting late CVE. Interestingly, the combination of high levels of proET-1 with high levels of IL-6 meant that the remaining inflammation showed the strongest risk of cardiovascular complication after 30 days and one year after CAP diagnosis [12].

### 2.5. Others

There are other biomarkers that have been evaluated. Carboxy-terminal provasopressin (CT-proAVP), known as copeptin, could also be useful for prognostic prediction [19,25,47,48]. Despite this, its use should be performed with caution because some factors such as antibiotic pre-treatment may influence its concentration in CAP patients [49]. There are no studies that evaluate their predictive role in CVE. Finally, Gutbier et al. published an interesting article demonstrating that angiopoietins, markers of endothelial barrier function and hyperpermeability, are involved in the pathogenesis of CAP and their quantification were useful in predicting short-term mortality [104].

## 3. Non-Cardiac Biomarkers

### 3.1. Inflammatory

#### 3.1.1. C-Reactive Protein

The C-reactive protein (CRP) is an acute phase protein widely used as a marker of systemic inflammation due to its high sensitivity and early elevation in response to acute damage, but it has low specificity as an infectious biomarker. In CAP, several studies have analysed the usefulness of CRP for diagnosis, severity assessment, and prognostic information [50,51]. Its utility for etiological diagnosis is controversial. Some studies showed high CRP levels in bacterial CAP in comparison to viral CAP [110,111,112]. However, CRP does not allow for optimal discrimination between viral and bacterial aetiology and has no role for treatment purposes. High levels of CRP at admission have been linked to an increased risk of complications and short-term mortality [52,53]. Its combination with PSI and CURB65 scores improves the 30-day short-term mortality prediction [11]. In addition, elevated levels maintained at 3–4 days are related to treatment failure and 30-day mortality [53,54] Regarding pneumonia diagnosis, CRP determination is not specific enough [55,56]. In fact, current European Respiratory Society and American Thoracic Society guidelines do not recommend systematic CRP determination for diagnoses or to guide treatment decisions [113,114], but it could be useful to diagnosis CAP in patients with atypical signs and symptoms or comorbid conditions, which could make other diagnoses more probable [115]. In any case, the correct interpretation of CRP levels can be a complicated issue. Factors, such as the time elapsed since the onset of symptoms, influence CRP kinetics. CPR is more useful in evaluating the inflammatory response of patients at three or more days since the onset of symptoms, because, within the first 48 h, their levels are still raising [116]. Finally, CRP may be useful for cardiovascular risk assessment in healthy women [117]. However, as recently was published, increased CRP levels failed to predict the occurrence of CVE in the short or long-term in CAP patients [12].

#### 3.1.2. Procalcitonin

Procalcitonin (PCT) is the active precursor of the hormone calcitonin belonging to the genetic CALC-1 family [57]. While it is mainly useful for detecting sepsis, it is also elevated in other acute inflammation situations such as pancreatitis, appendicitis, burns, polytrauma, and surgery. Interferon gamma, a molecule synthesized in the inflammatory immune response against viral infection, inhibits its synthesis, and therefore their levels are low in these infections. Due to its rapid kinetic in CAP patients within the first 48 h since the onset of CAP symptoms, PCT levels are more useful than CRP that is still mounting [116].

In contrast to other inflammatory biomarkers such as CRP, PCT has been considered more specific for bacterial infection. However, there is no PCT threshold that totally discriminates against viral and bacterial infection in CAP [9]. In recent studies, the ability of PCT to discriminate between viral and bacterial infection has been questioned. Despite higher levels being found in patients with severe pneumonia, PCT may not be elevated if CAP is caused by *Legionella pneumophila* or *Mycoplasma pneumoniae* and in post-influenza or mixed CAP. For this reason, PCT is not accurate enough to be used to determine whether empiric antibiotic therapy for CAP can be withheld because of a presumptive viral pathogen. PCT has been proposed to reduce the use of antibiotics and their duration [58,59]. Nevertheless, the last available studies have not shown that the determination of PCT involves a reduction in antibiotic consumption [60,61].

PCT is also useful as a prognostic biomarker. High levels of PCT at hospital admission for CAP are associated with ICU admission [62] and short-term mortality [10,63]. Furthermore, the persistence of elevated levels at 72 h before the start treatment is associated with treatment failure and mortality, while PCT being decreased improves the prognostic with a lower risk of shock, ICU admission, invasive mechanical ventilation, and death [54]. Finally, PCT has demonstrated a lack of utility for CVE prediction along, or in combination with, other biomarkers [12].

#### 3.1.3. Interleukin-6

Interleukin (IL)-6 is a classic proinflammatory cytokine. In the presence of microorganisms, lL-6 is produced immediately and contributes to host defence through the activation of the acute-phase inflammatory cascade and immune responses [64]. IL-6, IL-8, IL-10, interferon regulatory factor 5 (IRF5), and other inflammatory cytokines were found to be related with CAP severity, treatment failure and poor prognosis [65,66]. However, while a high IL-6 concentration was reported in typical bacterial pneumonia, this cytokine is not accurate enough for etiological purposes. IL-6 levels at days 1 and 3 were independently associated with a higher risk of treatment failure within the first 72 h or later [54]. Yende et al. have also reported that high systemic levels of IL-6 at hospital CAP discharge were associated with all-cause mortality over one year and cause specific mortality that is secondary to cancer, renal failure, infection or cardiovascular disease [67]. Menendez et al. have reported that high levels of IL-6, along with high levels of pro-ADM or proendothelin-1 at day 30, were associated with subsequent CVE (until a year after pneumonia)—as an expression of the deleterious effect of inflammatory persistence [12].

#### 3.1.4. Tumour Necrosis Factor Alpha

The tumour necrosis factor alpha (TNF-α) is an inflammatory mediator that activated a cellular immune response. Its effect is neutralized by a soluble anti TNF-α receptor. Unlike the inflammatory biomarkers seen previously, it is not studied in CAP. In contrast to CRP, PCT, and IL-6, TNF-α is unable to predict early or late treatment failure in the study by Menendez et al. and it does not improve mortality prediction [11]. However, reduced TNF-α levels are found in patients with low inflammatory systemic response, like COPD patients who developed community-acquired pneumonia [68]. The usefulness of this biomarker to assess cardiovascular risk in patients with CAP has not been analysed.

### 3.2. Non-Inflammatory

#### 3.2.1. Absolute Lymphocyte Count

Lymphocytes are the main actors in adaptive immunity. Absolute lymphocyte count (ALC), a biological marker that is cheap and widely available in all clinical settings, has been evaluated for mortality prediction in non-immunosuppressed CAP patients. Lymphopenia was shown to be an independent risk factor of 30 days mortality in two large cohorts of CAP patients [69]. Furthermore, the addition of this item to the CURB65 score improves its accuracy for mortality prediction. These findings have also been found in severe CAP (those admitted to ICU) and in ICU-acquired pneumonia [70,71]. Not only lymphopenia at diagnosis, but also persistent lymphopenia, are markers of poor prognosis [118]. Lymphopenia in CAP, mainly caused by a CD4^+^ depletion, has been found to be related to a dysregulated immune response with more inflammatory responses [72]. Lymphopenia could be caused by different reasons, including chronic diseases or critical illness, enhanced adhesion to the vascular endothelium, exhaustion, apoptosis or migration to the lungs [7,69]. ALC could also modify the treatment response to corticosteroids in severe CAP. In a post hoc analysis of a randomised clinical trial (RCT) that evaluated the effect of corticosteroids in patients with severe CAP and high inflammatory response, ALC may directly influence systemic inflammation reduction after treatment [73,119]. However, this effect should be prospectively evaluated in larger ad hoc RCTs.

#### 3.2.2. Neutrophil Extracellular Traps

Neutrophil extracellular traps (NETs) are webs formed by DNA, citrullinated histones, and proteases (including myeloperoxidase and neutrophil elastase) capable of snare and kill bacteria [74]. NETs are the result of neutrophil apoptosis (NETosis) in response to different stimuli. In some cases, this process is produced without the death of neutrophils, which preserve phagocytic activity, and are called vital NETosis [75]. Briefly, the triggers for NETs production include the interaction with pathogens [76], activated platelets [77], injured/activated endothelium [78], or reactive oxygen species (ROS) [79]. This interaction may be in the opposite sense and NETs can kill bacteria [74], activate platelets [80], damage the endothelium [81], and enhance inflammation [7].

The most data about NETs in infection mostly come from animal and in vitro models. There are only two studies in patients with CAP showing NETs as useful prognostic markers or as an endpoint in clinical trials [82,83]. In animal models, NETs have been shown to play a protector role against bacterial dissemination [84]. However, respiratory pathogens have developed evasion methods through the inhibition of release, degradation, and resistance to NETs [85,86]. It is believed that the excess availability of NETs in the tissues and in the bloodstream causes a harmful effect on the body. The main harmful components are the citrullinated histones and neutrophil proteases [87]. The deleterious effect of histones is due to several reasons. The histones have shown to be cytotoxic to lung epithelium and endothelium [88,89]. Another reason is that histones promote thrombosis [90]. This is due to several reasons: first, it is consequence of endothelial activation and damage [120]; and second, histones and NETs provide a scaffold for thrombi formation [91]. Finally, this causes cytotoxic consequences, ischemic events, and disseminated intravascular coagulation [92]. All this is capable of generating cardiovascular events, a concerning issue in this review. Previously, cardiotoxicity of histones during sepsis has been demonstrated [93]. Histone levels are directly correlated with cardiac troponin levels showing cardiac injury. Despite the data we have so far, there is much more needing to be in this area.

To date, the gold standard marker of NETosis or method of NET detection has not been determined [94]. The current methods or markers are as follows: co-localization of neutrophil-derived proteins and extracellular DNA, presence of citrullinated histones, cell-free DNA, myeloperoxidase/neutrophil elastase-DNA complex, image-based flow cytometric detection of NETosis, and flow cytometric detection of cell-appendant NET components. The differences lie in specificity, objectivity, and quantitativity, with advantages and disadvantages in each one. This adds complexity and hinders the translational use of these biomarkers today.

#### 3.2.3. Others

There are other biomarkers that have shown to be useful in pneumonia, but their use still requires further clinical studies. D-dimer, a product derived from fibrin degradation, also has prognostic implications. Low D-dimer levels have low-risk for short-term mortality in CAP patients [95,96], and elevated D-dimer levels are associated with poor outcomes, independently of clinical scores [97]. However, the adrenal function can also influence CAP outcomes [98]. Therefore, cortisol, as a marker of adrenal function, has demonstrated that it may help in prognosis prediction [99,100,101]. In addition, its measurement could identify the response of patients to steroids [102]. Meanwhile, high levels of the fibroblast growth factor 21 (FGF21) have recently been related to poor short-term prognosis in CAP patients from two randomised clinical trials [121]. FGF21 is a metabolic regulator with anti-inflammatory and immunoregulatory properties [103]. Siljan et al. recently evaluated the role of another biomarkers in CAP [10]. Presepsin, calprotectin, and pentraxin 3 (PTX3) are a soluble CD14-subtype molecule (sCD14-ST), a neutrophil biomarker, and a receptor involved in innate immunity, respectively. The study showed the potential use of calprotectin for bacterial aetiology discrimination and long-term prognosis, and a potential use of both presepsin (sCD14-ST) and PTX3 for short-term prognosis [10,122].

## 4. Limitations and Future Perspectives

While the study of CAP biomarkers has evolved tremendously over the last 25 years, many limitations remain and the search for the ideal biomarker is still open. In fact, the pathogenesis of CAP is an important topic for diagnosis, treatment, and the role of biomarkers. Biomarkers provide information about CAP from different perspectives: severity assessment, host’s immune-inflammatory response, organ failure, cardiovascular complications, and outcome. CAP is a complex disease with a heterogeneous host response depending on multiple factors such as the initial severity, comorbid conditions, and causal microorganism. Thus, this complexity is one of the biggest limitations. Each biomarker may reflect a specific pathophysiological response, but it will hardly cover all the perspectives that occur in pneumonia. For severity assessment, biomarkers improve the prognostic capacity of the scales, however, by themselves, they are not superior to them. Secondly, the technical difficulty in measuring some and the economic cost of others in many cases restricts their clinical use. Another limitation is time and kinetics. Biomarkers are not static and their measurement at a specific time does not give us a complete picture of the condition. Finally, the prior host status, severity, time since onset of infection or other factors can also influence biomarkers [111,116]. Thus far, despite all the information provided by biomarkers, clinical outcomes have not improved. We need more clinical trials with biomarkers involved in the design. This explains both the limited routine use of CAP biomarkers in clinical settings and the limited inclusion in the guidelines today. Nonetheless, biomarkers have their use. The potential utility of cardiac biomarkers has demonstrated, both in patients with previous cardiac disease and in those without [12]. They may guide the design of personalized cardiovascular rehabilitation programs and treatment optimization in patients both with and without known prior cardiac disorders.

The study of biomarkers must be addressed to achieve a more personalized medicine. Regrettably, the perfect biomarker does not exist at the moment. From our humble point of view, an interesting option could be to use a panel of biomarkers that depend on the outcome for evaluation. CRP is cheap, available and useful in monitoring responses, while PCT has a role in antibiotic duration. We consider that PCT is more useful during the first 2–3 days from the onset of symptoms, after this time, CRP seems to be better. Both the PCT and the CRP can be used together with the ALC for short-term survival prediction. For cardiovascular risk, proBNP, proADM, endothelin or troponins, offer similar information. Therefore, as Waterer et al. advocate, the future lies in embracing complexity [123]. In the coming years, it is very likely that the study of *omics* will bring new biomarkers in order improve the understanding and management of CAP. To this end, basic science has a determining role. Undeniably, a translational approach that combines basic and clinical science in a single effort will be essential in order to take steps in the right direction.

## Figures and Tables

**Table 1:** Main cardiac and non-cardiac biomarkers in community-acquired pneumonia patients.

Biomarker	Main Pathophysiological Mechanisms Involved	Main Potential Clinical Uses in CAP	Usefulness for Cardiovascular Risk Assessment in CAP	Main References
**Cardiac Biomarkers**				
Troponins	Myocardial injury	Short-term survival Long-term survival	Yes	[12,13,14,15,16]
Natriuretic peptides	Body fluid volume Cardiac overload/stress	Short-term survival Long-term survival	Yes	[12,17,18,19,20,21,22,23,24,25,26,27,28]
MR-proADM	Vasodilatation Inflammation	ICU admission Short-term survival Long-term survival	Yes	[12,28,29,30,31,32,33,34,35,36,37,38,39,40]
Endothelin-1	Vasoconstriction	Short-term survival Long-term survival	Yes	[12,17,41,42,43,44,45,46]
Copeptin	Body fluid volume Vasoconstriction	ICU admission Short-term survival Long-term survival	NA	[19,25,47,48,49]
**Non-Cardiac Biomarkers**				
CRP	Inflammation	Short-term survival	No	[11,12,50,51,52,53,54,55,56]
PCT	Infection	Etiological diagnosis ICU admission Short-term survival	No	[9,10,12,57,58,59,60,61,62,63]
IL-6	Inflammation	Short-term survival Long-term survival	No	[12,64,65,66,67]
TNF-α	Inflammation	No	NA	[11,68]
ALC	Adaptive immune response	Short-term survival Treatment response to corticosteroids	NA	[69,70,71,72,73]
NETs	Infection Inflammation Platelet activation Endothelial injury	Short-term survival	NA	[74,75,76,77,78,79,80,81,82,83,84,85,86,87,88,89,90,91,92,93,94]
D-dimer	Thrombus degradation	Short-term survival	NA	[95,96,97]
Cortisol	Adrenal function	Short-term survival Treatment response to corticosteroids	NA	[98,99,100,101,102]
FGF21	Metabolism regulation Inflammation Immune regulation	Short-term survival	NA	[103]
Calprotectin	Neutrophil inflammation	Bacterial aetiology discrimination Long-term survival	NA	[10]
Presepsin	Bacterial recognition	Short-term survival	NA	[10]
PTX3	Innate immune response	Short-term survival	NA	[10]
Angiopoietins	Endothelial barrier function	Short-term survival	NA	[104]

ALC: absolute lymphocyte count; CAP: community-acquired pneumonia; CRP: C-reactive protein; FGF21: fibroblast growth factor 21; ICU: intensive care unit; IL-6: interleukin-6; MR-proADM: midregional-proadrenomedullin; NA: not assessed; NETs: neutrophil extracellular traps; PCT: procalcitonin; PTX3: pentraxin 3; TNF-α: tumour necrosis factor alpha.

**Table 2:** Cardiac biomarkers, type of studies, design, and outcomes are evaluated.

Biomarker	Studies	Design	Number of Participants	Outcomes
Cardiac Biomarkers				
Troponins	Menéndez R et al. [12]	Prospective, observational and multicentre in CAP	730	Short-term cardiovascular events
	Bessière F et al. [13]	Meta-analysis in sepsis	1227	Short-term mortality
	Vallabhajosyula S et al. [14]	Retrospective in severe sepsis and septic shock	944	Short and long-term mortality
	Lee YJ et al. [15]	Retrospective in severe pneumonia	152	ICU mortality
	Vestjens SMT et al. [16]	Post hoc analysis of a clinical trial on adjunctive dexamethasone treatment in CAP	295	Short and long-term mortality
Natriuretic peptides	Menéndez R et al. [12]	Prospective, observational and multicentre in CAP	730	Short and long-term cardiovascular events
	Claessens Y-E et al. [18]	Prospective, observational and multicentre in mild CAP	549	Guide site of care
	Kruger S et al. [19]	Prospective, observational and multicentre in CAP	1740	Short and long-term mortality
	Christ-Crain M et al. [20]	Prospective, observational and single-centre in CAP	302	Treatment failure and short-term mortality
	Lin S-C et al. [21]	Prospective, observational and single-centre in severe CAP	216	Short-term mortality
	Chang CL et al. [22]	Prospective, observational and bicentric in severe CAP	474	Short-term mortality
	Akpinar EE et al. [23]	Prospective, observational and single-centre in CAP	179	ICU admission and short-term mortality
	Nowak A et al. [24]	Prospective, observational and single-centre in CAP	341	Short and long-term mortality
	Kruger S et al. [25]	Prospective, observational and multicentre in CAP	589	Short-term mortality
	Viasus D et al. [26]	Systematic review and meta-analysis	10319	Short-term mortality
	Alan M et al. [27]	Prospective, observational and multicentre in CAP	925	Long-term mortality
	Kruger S et al. [28]	Prospective, observational and multicentre in CAP	728	Short and long-term mortality
MR-proADM	Menéndez R et al. [12]	Prospective, observational and multicentre in CAP	730	Short and long-term cardiovascular events
	Kruger S et al. [28]	Prospective, observational and multicentre in CAP	728	Short and long-term mortality
	España PP et al. [34]	Prospective, observational and single-centre in CAP	491	Guide site of care
	Renaud B et al. [35]	Prospective, observational and multicentre in CAP	877	ICU admission
	Christ-Crain M et al. [36]	Prospective, observational and single-centre in CAP	302	Short-term mortality
	Huang DT et al. [37]	Prospective, observational and multicentre in CAP	1653	Short-term mortality
	Albrich WC et al. [38]	Prospective, observational and multicentre in LRTI	1359	Composite outcome: ICU admission, short-term mortality, and complications
	Liu D et al. [39]	Systematic review and meta-analysis	4119	Short-term mortality
	Bello S et al. [40]	Prospective, observational and multicentre in CAP	228	Short-term mortality
Endothelin-1	Menéndez R et al. [12]	Prospective, observational and multicentre in CAP	730	Short and long-term cardiovascular events
	Schuetz P et al. [44]	Prospective, observational and single-centre in CAP	281	ICU admission and short-term mortality
	Schuetz P et al. [45]	Prospective, observational and multicentre in CAP and LRTI	925	Composite outcome: ICU admission, short-term mortality, and complications
Copeptin	Kruger S et al. [19]	Prospective, observational and multicentre in CAP	1740	Short and long-term mortality
	Kruger S et al. [25]	Prospective, observational and multicentre in CAP	589	Short-term mortality
	Masia M et al. [47]	Prospective, observational and single-centre in CAP	173	Short-term mortality
	Kolditz M et al. [48]	Prospective, observational and single-centre in CAP	51	Composite outcome: ICU admission, short-term mortality, and clinical instability

CAP: community-acquired pneumonia; ICU: intensive care unit; LRTI: lower respiratory tract infections; MR-proADM: midregional-proadrenomedullin.
